# Organisational coaching to improve workplace resilience: a scoping review and agenda for future research

**DOI:** 10.3389/fpsyg.2024.1484222

**Published:** 2024-11-06

**Authors:** Abongile Sipondo, Nicky Terblanche

**Affiliations:** University of Stellenbosch Business School, Stellenbosch, South Africa

**Keywords:** resilience, organisational coaching, resilience coaching, scoping review, coaching

## Abstract

In an increasingly demanding and pressured work environment, employee resilience is acknowledged as a critical element to navigate adversity. There has been increased focus and interest in studying the nature of resilience in the workplace, however the mechanisms of developing and sustaining resilience are still under debate. Coaching is a promising method organisations use to improve employee resilience and provides employees with support to deal with the challenging working environment. There has been significant interest in coaching for resilience in recent years, however there is no overarching, consolidated view on the nature and dynamics of resilience coaching. This scoping review seeks to fill this gap by making three contributions. Firstly, we present details on various types of coaching approaches used to improve resilience. Secondly, we review the specific coaching elements and processes that lead to improved resilience and finally, we provide an overview on the efficacy of resilience coaching interventions. We conclude this scoping review with a roadmap for future research to help position and strengthen organisational coaching as a pillar of resilience development. This scoping review followed a five-stage PRISMA-ScR methodology which entails formulating research questions; identifying studies; choosing studies; extracting and charting data; and summarising the findings.

## 1 Introduction

The importance of resilience in the workplace has been increasingly recognised, with literature highlighting it as a crucial quality for employees (King et al., 2016; Hartwig et al., 2020). Resilience involves effectively utilising resources and skills to mitigate the adverse effects of negative experiences (Vanhove et al., 2016). It plays a significant role in how individuals cope with workplace stressors and is a key factor in preventing outcomes like anxiety, burnout, and compassion fatigue (Rees et al., 2015). Additionally, resilience encompasses the ability to adapt and endure stress, making it vital in today’s challenging work environment (Schwartz, 2018).

Coaching has emerged as a method organisations use to enhance resilience, providing employees with development tools and frameworks (Turk and Saue, 2021; Ali and Aziz, 2018). Research shows that various coaching approaches positively influence resilience (Lawton-Smith, 2017), and there is growing interest in using coaching to support employees facing tough work conditions (Stark, 2021). Evidence indicates that coaching effectively enhances resilience (Gyllensten and Palmer, 2012; Moore and Jackson, 2014), with specific interventions helping leaders navigate challenging work environments (Bennett and Lemoine, 2014). Studies by Grant et al. (2009) and Sherlock-Storey et al. (2013) demonstrated improvements in resilience following coaching interventions.

However, a significant gap in coaching research is the lack of studies examining the processes of change within coaching (Grover and Furnham, 2016), particularly in resilience coaching. Despite growing interest in this area, no systematic or scoping reviews specifically addressing coaching interventions aimed at increasing psychological resilience in the workplace. To address this gap, we conducted a scoping review of resilience coaching interventions, following a five-stage PRISMA-ScR methodology (Arksey and O’Malley, 2005). The review was guided by three research questions:

1. What coaching approaches are currently used in resilience coaching?
2. What processes are followed in resilience coaching?
3. What benefits do participants derive from resilience coaching?

This scoping review fills an evident gap in the literature by systematically compiling effective resilience coaching interventions, critically examining various coaching approaches, and deepening the understanding of the dynamic processes through which resilience develops in coaching. The findings aim to help coaches better design interventions that promote resilience in the workplace (Liu et al., 2019).

## 2 Conceptual and theoretical perspectives

### 2.1 Psychological resilience

The terminology surrounding psychological resilience lacks consistency, with various terms used interchangeably, such as personal resilience, mental resilience, emotional resilience, cognitive resilience, and individual resilience (IJntema et al., 2019). Scholars have offered different definitions of psychological resilience. Luthar et al. (2000) describe it as “a dynamic process encompassing positive adaptation within the context of significant adversity” (p. 20). Meanwhile, Paul and Garg (2014) define resilience as “a unique ability to endure and recover fully from extreme conditions, setbacks, trauma, and other adversity” (p. 72).

The inconsistency in the definition of resilience is the difference in conceptualisation of resilience by different researchers. Resilience is a complex concept, and research has conceptualised it as a trait, an individual’s skills or abilities, or a capacity to function positively when exposed to adversity (Van Breda, 2018). Early studies suggested that resilience was characterised by static character traits, with the trait-oriented approach focusing on a hardy personality type (Chmitorz et al., 2018; Galazka and Jarosz, 2019). Later, it was viewed as an outcome, where psychological health is maintained or recovered despite challenges (Kalisch et al., 2017). The outcome-oriented approach emphasises psychological health being sustained or recovered despite adversity (Southwick et al., 2014). Resilience is flexible and influenced by various factors, including internal factors like genetics and resilience-conducive personality traits (Bonanno and Diminich, 2013; Masten, 2001). In this article, we align our conceptualisation of resilience with the emerging view in recent research suggests that resilience can also be viewed as a dynamic and interactive process (Southwick and Charney, 2012). This view is supported by literature indicating that resilience is a malleable epiphenomenon that can be developed, and that individuals can learn to deal with adversity (Neenan, 2018; Winwood et al., 2013; Liu et al., 2019).

### 2.2 Benefits of increased resilience in the workplace

Research underscores the significance of enhancing psychological resilience among employees, especially during organisational change, as it can protect against negative impacts (Brown and Abuatiq, 2020). Building resilience is associated with reduced burnout and its effects (Ghossoub et al., 2020) and contributes to improved mental health, positive emotions, self-efficacy, and coping skills (Ke et al., 2022).

The benefits of psychological resilience include greater wellbeing, higher self-efficacy, increased job satisfaction, and improved productivity (McEwen and Boyd, 2018). Resilient individuals experience less anxiety, demonstrate cognitive flexibility, and are more likely to view challenges positively (Baker et al., 2021). Other advantages include a sense of control, effective coping, and personal development opportunities (Fletcher and Sarkar, 2013). Overall, individuals with high resilience approach life with optimism and energy, making them less vulnerable to stress-related issues like depression and burnout (Young, 2014).

### 2.3 Coaching for resilience

Research emphasises the growing emphasis on resilience coaching within research and practice, highlighting its effectiveness in helping individuals navigate challenges in the workplace. Lawton-Smith (2015) suggests that coaching serves as a proactive strategy for enhancing psychological resilience. A meta-analysis by Vanhove et al. (2016) finds that coaching is more effective than traditional classroom-based approaches for building resilience. Coaching offers distinct advantages, such as providing a confidential space for discussing difficulties, proactively developing skills, and facilitating open conversations about challenges. Research indicates that coaching empowers individuals to manage their professional lives and make informed career choices (Dyrbye et al., 2019).

In the context of workplace coaching, the coach assists the coachee in developing a self-regulation process that enhances their wellbeing (Fontes and Dello Russo, 2021). Bozer and Jones (2018) describe workplace coaching as a personalised, collaborative intervention aimed at achieving the coachee’s goals. Coaches create a supportive environment that encourages self-reflection, poses challenging questions, and work with coachees to devise solutions and action plans (Grant, 2014). This guided introspection fosters self-awareness, enhances self-control, and alleviates anxiety (Grant, 2017). Reflecting on one’s strengths and weaknesses cultivates resilience-related skills, such as improved coping mechanisms and problem-solving abilities (Grant and Kinman, 2014).

## 3 Methods

Scoping review guidelines (Arksey and O’Malley, 2005, PRISMA-ScR: Tricco et al., 2018) were followed in conducting this scoping review. The five stages of scoping review methodology, as outlined by Arksey and O’Malley (2005) were applied as follows: (1) formulating research questions; (2) identifying studies; (3) choosing studies; (4) extracting and charting data; and (5) summarising the findings.

### 3.1 Identification of studies

The Stellenbosch University multi-database search engine, Ebscohost, Scopus, and Web of Science were all targeted in a comprehensive search strategy that was developed using text words found in the titles and abstracts of pertinent papers as well as the index keywords used to describe the articles. Each identified keyword and index terms in the search strategy was modified for each database and/or information source that was used. We created an appropriate research string for each database by combining the terms “coaching for resilience,” “resilience coaching,” and “resilien∗ AND coach∗,” and we searched inside titles, abstracts, and keywords.

### 3.2 Inclusion and exclusion criteria

The review examines psychological resilience coaching for adults in workplace settings. The search criteria included only peer-reviewed journal articles in English with available abstracts. In addition to database searches, the researchers employed a snowballing technique, exploring the reference lists of existing reviews and identified publications.

Studies were excluded based on several criteria, including:

- Non-peer-reviewed articles (e.g., book reviews, conference papers, theses, and dissertations).
- Focus on types of resilience other than psychological resilience.
- Non-adult populations.
- Publications in languages other than English.
- Textbooks, opinion pieces, or practitioner contributions lacking empirical data.
- Editorials or philosophical papers.
- Articles with substantial content overlap or exact duplicates.
- Non-accessible articles (i.e., those that were not open access or could not be accessed via the university library).

### 3.3 Selection of studies

After the search for relevant studies, duplicate citations were removed, and the remaining references were uploaded into EndNote 21/2023. Three reviewers convened to discuss the inclusion and exclusion criteria for studies, as outlined by Levac et al. (2010). Each reviewer independently assessed the abstracts from the search, refining the search strategy based on their findings.

**Figure 1:**
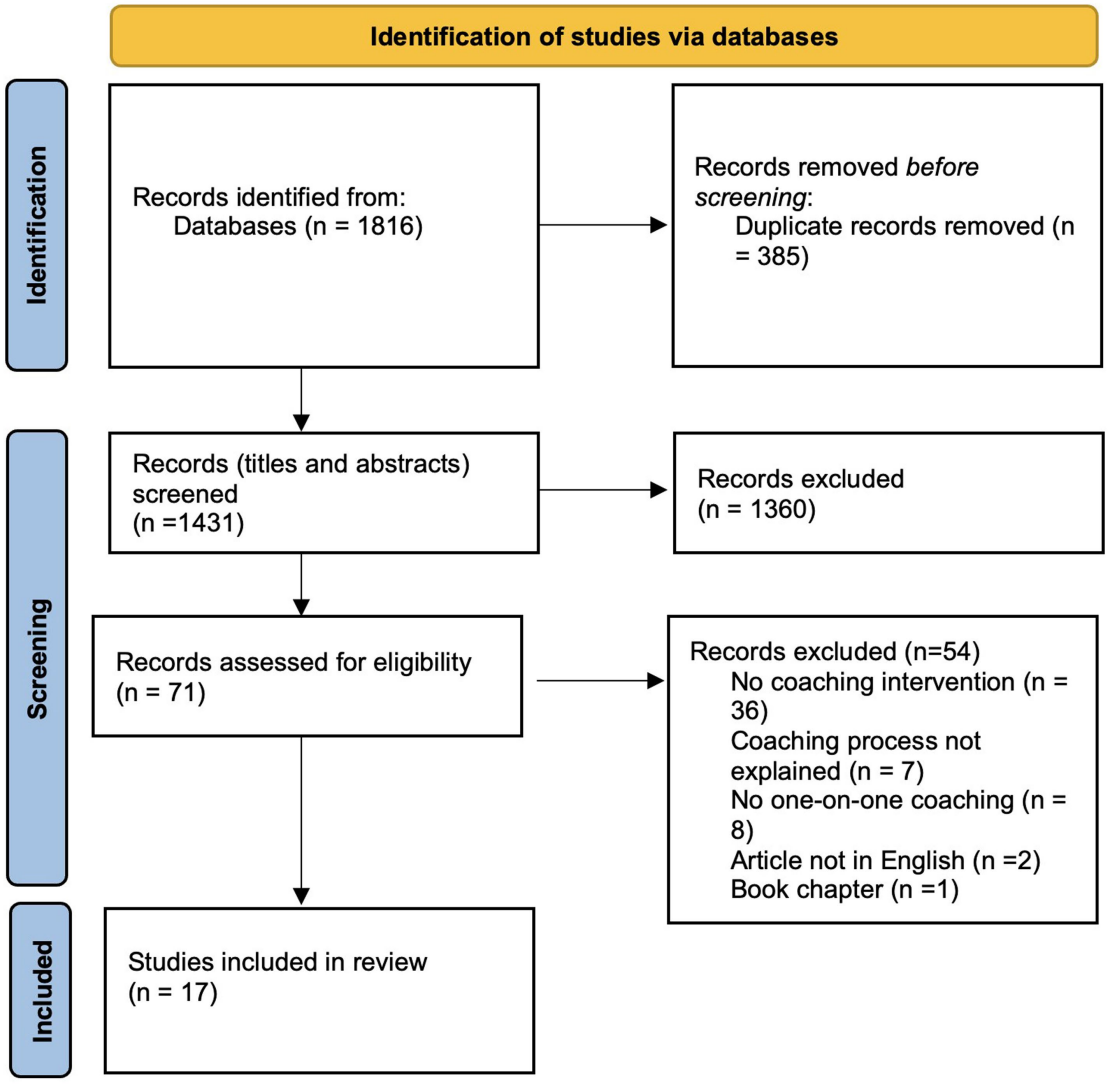
Paper selection process.

We then reviewed full articles for inclusion, retrieving texts for potentially relevant studies and updating EndNote with citation details. The reviewers evaluated the complete texts against the inclusion criteria and resolved any disagreements through discussion. The outcomes of the search and selection process are illustrated in a PRISMA-ScR flow diagram (Tricco et al., 2018), shown in Figure 1.

### 3.4 Data extraction

A comprehensive set of information on the participants, concept, context, study methods, and significant findings pertinent to the review question are included in the extracted data. An Excel spreadsheet was used to keep a systematic data-extraction as well as analysis process. We extracted the pertinent data from each study, including the authors, year, study design, sample, and results. This phase gave a broad summary of resilience coaching programme elements from the literature and served as the basis for a more in-depth analysis.

### 3.5 Data analysis and presentation

In this scoping review, we employed qualitative content analysis to identify key qualities and factors associated with resilience coaching. This method, following the framework established by Kleinheksel et al. (2020), involved using frequency counts to assess the prevalence of specific codes within the text as an indicator of their significance. The analysis process began with an immersive reading of the text, alongside transcribing recorded data, which allowed us to become intimately familiar with the material and generate preliminary ideas about potential concepts or themes (Kleinheksel et al., 2020). The first step involved identifying units of meaning within the text. After recognising and condensing these units, we assigned codes to them, organizing the data for greater clarity. Next, we used two or more code categories to uncover or support broader underlying meanings, ultimately leading to the development of themes (Kleinheksel et al., 2020). This thorough examination of each publication was conducted over multiple rounds to ensure a comprehensive understanding of the material.

## 4 Results

### 4.1 General overview

We found 17 relevant papers, with a focus on resilience coaching interventions in business settings (see Table 1). The findings are presented in a narrative and tabular format, summarising the resilience coaching programmes, their characteristics, and the outcomes.

**Table 1:** Summary of included studies.

Authors and date of publication	Study design	Sample size	Number of sessions and duration of coaching programme	Coaching approach	Outcome measures	Data analysis	Measurement time points	Outcomes
Archer and Yates (2017)	Interpretative phenomenological analysis.	5	Four coaching sessions in 6 months	Positive psychology.	Semi-structured interviews a month after the intervention.	Qualitative—thematic analysis.	Two time points—Pre-and Post measures.	Increased resilience.
Auer et al. (2022)	Mixed quasi-experimental design.	1,005	3–45 coaching sessions in 6–40 weeks.	Not specified.	A single Likert-type item “I recover quickly after stressful experiences.”	Quantitative - series of linear mixed-effect models with random intercepts and fixed predictors.	Two time points—Pre-and Post measures.	Increased resilience in the coaching group.
Brown and Yates (2018)	Action research study.	5	Three sessions in 3 months.	Humanistic approach drawing on Positive Psychology, solutions focused and cognitive behavioural.	Semi-structured interview.	Qualitative - Interpretative Phenomenological Analysis.	Qualitative data (1 time point).	Increased resilience.
De Haan et al. (2019)	Randomised study design.	180	Maximum 12 sessions in 14 months.	Not specified.	Brief Resilience Scale.	Quantitative—*T*-tests.	Three Time Points (Pre-, Post-, and Follow-Up Measures).	Coaching increased resilience
Dyrbye et al. (2019)	Pilot randomised clinical trial.	82	Five coaching sessions in 5 months.	Not specified.	10-item Connor-Davidson Resilience Scale.	Quantitative - SAS, Kruskal-Wallis or *χ*^2^ tests	Two time points—Pre-and Post measures.	Intervention group increased resilience.
Fontes and Dello Russo (2021)	Experimental field study.	56	Four sessions once a month.	Not specified.	Psychological Capital Questionnaire short form (PCQ-12).	Quantitative - ANOVA with repeated measures.	Three time points (Pre-, Post-, and Follow-Up Measures).	Increase in PsyCap.
Grant et al. (2009)	Randomised controlled waitlist design.	41	Four sessions in 8–10 weeks.	Cognitive behavioural, solution focused framework.	18-item Cognitive Hardiness Scale.	Mixed methods- repeated measures ANOVA; thematic analysis (Spector, 1984).	Three time points (Pre-, Post-, and Follow-Up Measures).	Enhanced resilience.
Grant et al. (2010)	Mixed experimental and quasi-experimental designs.	50	10 sessions in 20 weeks.	Cognitive behavioural, solution focused framework.	18-item Cognitive Hardiness Scale.	Quantitative—repeated measures ANOVA.	Three time points (Pre-, Post-, and Follow-Up Measures).	The coaching group reported increased resilience.
Grant (2014)	Within-subjects (pre2post) design.	31	Four sessions.	Cognitive behavioural, solution focused framework.	10-item Cognitive Hardiness Scale.	Mixed methods—Paired *t*-tests; Open-question method.	Two time points—Pre-and Post measures.	The coaching programme was effective at enhancing resilience.
Grant et al. (2017)	Within-subjects (pre2post) design.	31	Six sessions in 6 months.	Cognitive behavioural, solution focused framework.	10-item cognitive hardiness scale	Mixed methods—Paired *t-*tests; Open-question method.	Two time points– pre-and post measures.	Participants become more resilient.
Gray (2016)	Empirical phenomenological case study.	4	Four sessions	Custom wellbeing coaching model based on positive psychology, neuroscience, and pedagogy.	7 item Likert scale.	Qualitative - empirical phenomenological case study.	Three time points (pre-, post-, and follow-up measures).	Participants experienced a transformational understanding of individual resilience at work. Construction of new schema relating to resilience.
IJntema et al. (2021)	Quasi-experimental field study.	91	Four sessions in 13 weeks.	Not specified.	6-item brief resilience scale.	Quantitative - SPSS 24	Three time points (Pre-, Post-, and Follow-Up Measures).	Positive immediate and long-term effects were found.
Jeannotte et al. (2021)	Longitudinal, observational within-subjects design.	391	At least eight coaching sessions.	Not specified.	Custom nine dimensions scale were newly developed for BetterUp.	Mixed study - self-report survey measures; questionnaires; ANOVA.	Three time points (pre-, post-, and follow-up measures).	Increased resilience. Larger resilience gains in the second half of the intervention (*β* = 0.08–0.18).
McKimm and Povey (2018)	Mixed-methods study.	52	Between 3 months and 1 year.	Not specified.	Robertson Cooper i-resilience online tool.	Mixed methods - survey questionnaire; semi-structured interviews; human function curve; Robertson Cooper’s validated online i-resilience questionnaire	2 time points– Pre-and Post measures.	Participants moved from “distress,” “boredom” and excess pressure nearer to the “safe zone.”
Sherlock-Storey et al. (2013)	Test/re-test design.	52	Three coaching sessions over 6 weeks.	Not specified.	Psychological capital (PsyCap) questionnaire PCQ.	Quantitative - Kolmogorov-Smirnoff tests, sample *t*-tests.	2 time points– pre-and post measures.	Increased personal resilience.
Song et al. (2020)	Mixed-methods study.	25	Eight sessions over an academic year.	Positive psychology approach.	6-item brief resilience scale.	Qualitative - reformulated grounded theory.	2 time points—pre-and post measures.	Improved resilience.
Timson (2015)	Qualitative.	6	Not specified.	Not specified.	Thematic analysis.	Qualitative—thematic analysis.	Qualitative data (1 time point)	Coaching provided time and space for reflection and learning.

**Figure 2:**
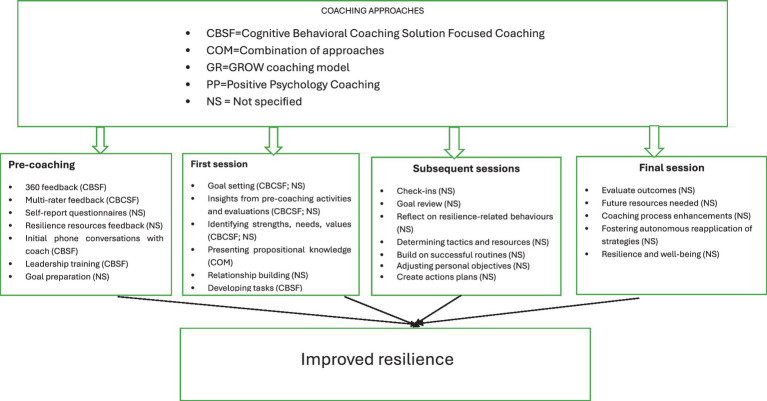
Summary of the coaching approaches and steps.

Figure 2 summarises the steps involved in the coaching sessions, linking the steps to approaches aimed at fostering resilience, elucidating how these approaches were measured and how their effectiveness was evaluated.

### 4.2 Coaching approach

The reviewed studies employed various coaching approaches, with the most common being the cognitive behavioural and solution-focused framework, used in four studies (Grant et al., 2009; Grant et al., 2010; Grant, 2014; Grant et al., 2017). Two studies utilised a positive psychology approach (Archer and Yates, 2017; Song et al., 2020). Additionally, two studies combined multiple approaches. Brown and Yates (2018) applied a humanistic approach, integrating elements from positive psychology, solution-focused coaching, and cognitive behavioural coaching. Gray (2016) created a wellbeing coaching approach for individuals and teams facing organisational transitions, drawing on positive psychology, neuroscience, and pedagogy. However, nine studies (Auer et al., 2022; Dyrbye et al., 2019; De Haan et al., 2019; Fontes and Dello Russo, 2021; IJntema et al., 2021; Jeannotte et al., 2021; McKimm and Povey, 2018; Sherlock-Storey et al., 2013; Timson, 2015) did not specify the coaching approach used. Lastly, three studies (Fontes and Dello Russo, 2021; Grant et al., 2009; Grant et al., 2010) employed the GROW model (Goal, Reality, Options, Way forward) to ensure the coaching conversations were goal-oriented and structured.

### 4.3 Coaching delivery steps

#### 4.3.1 Pre-coaching activities

In eight studies, participants engaged in various pre-coaching exercises before the first coaching session (Auer et al., 2022; De Haan et al., 2019; Fontes and Dello Russo, 2021; Grant et al., 2009; Grant et al., 2010; Grant et al., 2017; IJntema et al., 2021; Sherlock-Storey et al., 2013). These activities included:

- Assessments (IJntema et al., 2021; Jeannotte et al., 2021)
- 360-degree feedback (Grant et al., 2009)
- Multi-rater feedback (Grant et al., 2010)
- Initial phone conversation with their coach (Grant et al., 2017)
- Online questionnaires (De Haan et al., 2019)
- Self-report questionnaire (Fontes and Dello Russo, 2021)
- Pre-COVID-19 surveys (Auer et al., 2022)
- Pre-coaching activities (Sherlock-Storey et al., 2013)

In six studies (Archer and Yates, 2017; Grant et al., 2009, 2010, 2017; IJntema et al., 2021; Sherlock-Storey et al., 2013), additional activities beyond assessments were included. For example:

- In Grant et al. (2009), participants received 360-degree feedback on their leadership styles and attended a half-day leadership training course.
- In Grant et al. (2017), participants had an introductory phone conversation with their selected coach.
- In IJntema et al. (2021), participants received individual feedback reports on their resilience resources before the first session.
- Archer and Yates (2017) collected an initial written description of participants’ career confidence prior to coaching.
- Grant et al. (2010) involved an orientation meeting before coaching.
- Sherlock-Storey et al. (2013) included pre-coaching activities where participants set preliminary goals related to resilience and wellbeing, with a workbook providing reflective exercises.

These preparatory activities helped participants reflect on their resilience, leadership styles, and personal goals, thereby enhancing the effectiveness of the coaching interventions.

#### 4.3.2 Typical coaching session

Seven studies discussed the roles of both the coach and coachee during coaching sessions (Dyrbye et al., 2019; Grant, 2014; Grant et al., 2009; Grant et al., 2010; Grant et al., 2017; Gray, 2016; Jeannotte et al., 2021), emphasizing their collaborative dynamic.

Coachee’s role:

- *Self-led development*: Coachees took the lead in conversations, steering their own growth and progress at their own pace (Jeannotte et al., 2021).
- *Schemata identification and replacement*: Participants identified and replaced harmful thought patterns through guided inductive reasoning and co-construction during coaching sessions (Gray, 2016).
- *Session preparation*: Before each session, coachees completed a readiness document, outlining their objectives, progress, and challenges faced (Grant, 2014).
- *Documentation of insights and actions*: Coachees were responsible for documenting personal insights and agreed-upon action plans during coaching (Grant, 2014).

Coach’s role:

- *Facilitating introspection and collaboration*: Coaches provided a private space for reflection, asked challenging questions, and collaborated with coachees to generate ideas, find solutions, and create action plans (Grant, 2014).
- *Monitoring progress*: Coaches guided coachees through the self-regulation cycle, helping them develop action plans, monitor progress, and evaluate it between sessions (Grant et al., 2009; Grant et al., 2017).
- *Self-reflection*: Coaches maintained self-reflection notebooks after each session to ensure they adhered to best practices and maintained the fidelity of the coaching program (Dyrbye et al., 2019; Grant et al., 2010).
- *Encouraging behaviour change*: At the end of each session, coaches helped create a list of specific action steps for the coachees to complete before the next session, aiming to facilitate meaningful behavioural changes (Grant, 2014).

This balance between the coachee’s proactive involvement and the coach’s structured guidance was key in fostering resilience and personal development.

#### 4.3.3 First coaching sessions

In four of the reviewed studies (Fontes and Dello Russo, 2021; Grant et al., 2009; IJntema et al., 2021; Sherlock-Storey et al., 2013), the insights from pre-coaching activities and evaluations were revisited and assessed during the first coaching session. According to four studies (Archer and Yates, 2017; Dyrbye et al., 2019; Grant et al., 2010; Sherlock-Storey et al., 2013), participants focused on identifying their strengths, needs, values, and areas of resilience during this initial session. This helped set a foundation for the coaching process by ensuring the participants had a clear understanding of their personal capacities and challenges. Goal setting was a key activity in the first session across six studies (Dyrbye et al., 2019; Fontes and Dello Russo, 2021; Grant et al., 2009, 2010; IJntema et al., 2021; Sherlock-Storey et al., 2013), where participants collaboratively established specific objectives that would guide their coaching journey.

In the Gray (2016) study, the first session was slightly different as it focused on presenting propositional knowledge about workplace stress and explaining the coaching model and procedure to the participants. This educational aspect ensured that participants had a clear understanding of how the coaching would proceed and what the expected outcomes might be.

In the Dyrbye et al. (2019) study, the goal of the first session was to build a relationship between the coach and coachee, while also developing an action plan. The establishment of trust and rapport was seen as a critical step in enabling successful coaching outcomes.

Finally, in two instances (Grant et al., 2009; Grant et al., 2010), coachees were tasked with developing between-session action steps during the first session. These tasks were designed to be completed before the next meeting, allowing participants to actively engage with their goals and make early progress in their development.

#### 4.3.4 Subsequent coaching sessions

In the reviewed studies, a variety of activities were noted to take place during subsequent coaching sessions:

1. *Checking in and reviewing progress*: Participants frequently reviewed any strategic actions they had taken since the previous session, monitored their progress towards set goals, and discussed accountability for their actions (Dyrbye et al., 2019; IJntema et al., 2021; Sherlock-Storey et al., 2013).
2. *Determining tactics and resources*: In some sessions, participants explored potential strategies and resources that could help them meet their objectives (Fontes and Dello Russo, 2021).
3. *Fostering resilience and self-efficacy*: Participants focused on enhancing their resilience by reflecting on and replicating successful episodes and routines, thereby building their self-efficacy (Fontes and Dello Russo, 2021).
4. *Adjusting personal objectives*: As participants progressed, they adjusted their personal goals as needed to reflect their evolving challenges and achievements (IJntema et al., 2021).
5. *Creating action plans*: Participants developed action plans during each session to guide their efforts in achieving goals and developing the necessary resources between sessions (IJntema et al., 2021).

These activities reinforced continuous reflection, adaptation, and accountability, all of which were integral to building resilience and achieving the desired outcomes in the coaching process.

#### 4.3.5 Between the coaching sessions

Two studies highlighted activities that coachees could engage in between coaching sessions:

1. In the Jeannotte et al. (2021) study, an algorithm based on the topic of the coaching session suggested various resources to the coach, which were then shared with the coachees. These resources included readings, audio or video content, guided or self-directed exercises, and other materials to support the coachees’ development between sessions.
2. In the IJntema et al. (2021) study, participants were required to complete two Psyfit modules as “homework” to enhance their resource-building efforts between coaching sessions. Coachees could choose from six different modules, including mastering life skills, improving self-esteem and relationships, practicing mindfulness, fostering positive thinking, and setting personal goals.

These activities were designed to extend the benefits of coaching beyond the sessions by encouraging continuous learning and self-development.

#### 4.3.6 Final coaching sessions

Four studies described the activities conducted in the final coaching sessions:

1. In the IJntema et al. (2021) study, participants evaluated their level of objective achievement, developed an action plan for future resource development, and assessed the overall coaching process.
2. The Fontes and Dello Russo (2021) study focused on evaluating participants’ progress, enhancing their self-confidence, and encouraging them to independently reapply the strategies they had learned during coaching.
3. In the final session, participants gained a clearer understanding of the resources available to them for building resilience, which was a central focus of the coaching program.
4. The Sherlock-Storey et al. (2013) study aimed to help participants plan for continued resilience and wellbeing maintenance without the need for ongoing coaching. Coachees were encouraged to set future goals extending beyond the coaching programme to ensure long-term development.

These final sessions were designed to ensure that participants could apply their learning independently, assess their progress, and create plans for continued growth and resilience.

### 4.4 Study design elements of the coaching interventions

The goals of the coaching programmes were diverse, with only a few studies focusing solely on resilience (IJntema et al., 2021; Sherlock-Storey et al., 2013). Other programmes aimed to improve wellbeing (Dyrbye et al., 2019), facilitate workplace transitions (Grant, 2014), or enhance leadership development (Grant et al., 2017). The methodologies used also varied, with experimental and quasi-experimental designs being most common, and data collected at different intervals, typically before and after the interventions, with follow-up measures in some cases. These differences in design, objectives, and data collection methods further contribute to the challenge of comparing the effectiveness of these coaching programmes.

Coaching interventions in the reviewed studies also showed significant variation in both duration and structure. The length of programmes ranged from 6 weeks (Sherlock-Storey et al., 2013) to over a year (McKimm and Povey, 2018), with some studies offering flexible timeframes (Auer et al., 2022; McKimm and Povey, 2018). The number of sessions also varied, from a minimum of three (Brown and Yates, 2018) to up to 45 sessions (Auer et al., 2022). Intervals between sessions ranged from weekly (Grant et al., 2010) to every 4–6 weeks (Archer and Yates, 2017), and session lengths fluctuated between 30 and 90 min, further highlighting the absence of a standardised approach across studies.

The delivery and expertise of the coaches also varied significantly. While most studies used external, certified professional coaches (Auer et al., 2022), some employed internal coaches (De Haan et al., 2019). The coaches’ qualifications ranged from short formal training programmes to PhDs, and their experience varied widely. This inconsistency in coach experience, along with differences in delivery methods (in-person, virtual, or hybrid), adds another layer of complexity to assessing the impact and success of coaching interventions across the reviewed studies. Standardising coach qualifications, delivery methods, and programme structure could enhance future research and practice in the field.

### 4.5 Outcomes of the coaching interventions

Participation in coaching has been shown to improve resilience in all the reviewed studies, with both qualitative and quantitative measures supporting these findings. A key theme across seven studies was that the intervention group, which received coaching, experienced greater resilience improvements compared to the control group (Auer et al., 2022; Dyrbye et al., 2019; Grant, 2014; Grant et al., 2009; Grant et al., 2010; Grant et al., 2017; Song et al., 2020). For instance, in Auer et al. (2022), those receiving coaching had significantly larger resilience gains than those without coaching. Fontes and Dello Russo (2021) found that coaching was associated with increases in Psychological Capital, while Grant et al. (2010) also reported increased resilience in the coaching group compared to the control. Similarly, Grant (2014) found that coaching enhanced resilience, with a one-tailed *t*-test showing significant increases [*t*_(1, 30)_ = 1.79, *p* < 0.05]. Grant et al. (2017) demonstrated that participation led to significant increases in resilience [*t*_(1, 30)_ = 2.50, *p* < 0.05]. In Sherlock-Storey et al. (2013), post-intervention resilience levels were significantly higher [*t*_(10)_ = 3.24, *p* = 0.045], while in Dyrbye et al. (2019), resilience scores improved more in the intervention group than the control group [mean (SD) = 1.3 (5.2) vs. 0.6 (4.0)]. Song et al. (2020) reported significant improvements in mean BRS scores, though the lack of a control group limited conclusions about the direct effects of the coaching intervention.

Qualitative findings also showed enhanced resilience following coaching (Archer and Yates, 2017; Brown and Yates, 2018; Grant et al., 2009; Gray, 2016; Timson, 2015). Brown and Yates (2018) identified emotional changes during and after coaching as a dominant theme. IJntema et al. (2021) found that resilience components like hope, self-efficacy, and stress recovery had both short- and long-term positive impacts. Gray (2016) reported a transformative awareness of resilience and workplace wellbeing, suggesting that new resilience-related schemata can be developed through cognitive and behavioural repetition. Archer and Yates (2017) observed deliberate shifts in participants’ thinking, leading to greater resilience and self-reflection. Timson (2015) highlighted the value of the individualised coaching sessions in fostering self-reflection and insight, which were key to developing behavioural changes. De Haan et al. (2019) also found correlations between resilience, psychological wellbeing, self-efficacy, and social support as indicators of coaching effectiveness. Jeannotte et al. (2021) noted that resilience development took longer, with the most significant gains occurring in the latter half of the intervention.

## 5 Discussion

The objectives of this scoping review were to examine:

- The coaching approaches are currently used in coaching for resilience,
- The processes that are followed in resilience coaching.
- The efficacy of the resilience coaching interventions

### 5.1 The coaching approaches that are currently used in coaching for psychological resilience

This scoping review has shown that various approaches have been successfully used to coach for resilience, including the positive psychology approach (Archer and Yates, 2017; Song et al., 2020) and a humanistic approach incorporating positive psychology, solution-focused, and cognitive-behavioural coaching (Brown and Yates, 2018). This is consistent with the research, wherein a number of academics have shown that resilience can be enhanced by various coaching strategies (Pemberton, 2015). However, the most used approach is the cognitive-behavioural coaching (CBC) framework (Grant et al., 2009, 2010; 2014; 2017), which focuses on identifying internal resources and overcoming negative thinking (Skews et al., 2018). Resilience is dependent on flexibility in thoughts and actions when reacting to adverse situations (Neenan, 2018). CBC helps coachees reframe irrational beliefs (David et al., 2016) and develop resilient emotions (Skews et al., 2018).

### 5.2 The coaching elements that need to be present in the psychological resilience coaching process

#### 5.2.1 Development of resilience resources

The creation of resilience resources by the coachees was another element that the scoping review emphasised. It is highlighted that coachees are cognizant of the resources at their disposal to cultivate resilience (Archer and Yates, 2017). Resilience is shaped by the availability of resilience-promoting resources (Joyce et al., 2018). Resilient individuals can effectively access current, latent, or new resources to overcome adversity (Young, 2014). Optimal resilience interventions focus on maximising personal resources to manage stress (Fletcher and Sarkar, 2016; Forbes and Fikretoglu, 2018). Access to resources helps mitigate stress and shields against its negative effects (Skews et al., 2018). Both external and internal resources, particularly psychological ones, play a key role in resilience (Luthans et al., 2006). Developing psychological resources, such as psychological capital, strengths, and mental toughness, is vital for enhancing resilience (Skews et al., 2018).

#### 5.2.2 Coach and coachee relationship

An additional component that was identified as crucial to the development of the coachee’s resilience was the coach-coach relationship. The coaching relationship plays a crucial role in fostering resilience by helping coachees expand their perspectives and interpret events more flexibly (Neenan, 2018). This aligns with research indicating that sustaining the coachee’s attention and connection between coaching sessions and managing relationships are crucial. The coachee could easily lose interest in the coach and the process without the relationship (Koortzen and Oosthuizen, 2010). Transformation and growth occur within the context of a strong coach-coachee relationship, which is a key variable in the coaching process (Mosteo et al., 2016). This relationship involves collaboration, setting clear objectives, and developing action steps for goal attainment (Grant et al., 2009). A supportive coaching relationship can alleviate stress and anxiety (Grant, 2014), and feedback from the coach reinforces positive outcomes and resilience (Fontes and Dello Russo, 2021).

#### 5.2.3 Homework between the coaching sessions

It is essential for coachees to engage in work between coaching sessions, often referred to as “homework,” to facilitate resource development (IJntema et al., 2021). Coaches should assist coachees in creating actionable steps to be completed between sessions (Grant et al., 2009). One effective method for this is to have coachees fill out a preparation sheet before each session (Grant, 2014). This paperwork allows participants to outline their goals for the upcoming sessions, document their progress, and identify specific challenges they have faced (Grant, 2014).

#### 5.2.4 Positive adaptation and appraisal of events is required to enhance resilience

The literature emphasises that positive adaptation is essential in building resilience (Van Breda, 2018). Resilience is linked to positive adaptation, recovery, and psychological growth, helping individuals thrive through adversity (Baker et al., 2021). Positivity during crises has been shown to foster resilience (Grant and Kinman, 2014; Ke et al., 2022), and resilience involves the processes that enable individuals to adapt positively (Britt et al., 2016). Resilience as a concept encompasses both the resources that support positive adaptation and the adaptive processes themselves (Galazka and Jarosz, 2019).

The way individuals appraise stressful events is a key factor in how they respond to adversity (Young, 2014). Resilience goes beyond coping and recovery, influencing both event appraisal and one’s capacity to manage the adverse event (Baker et al., 2021). As a process, resilience involves adapting based on feedback and experiences (Liu et al., 2019), with success often determined by how individuals respond to challenges, not just the challenges themselves (Jackson and Watkin, 2004). Attitude plays a central role in resilience (Palmer, 2013), and when confronting stress is ineffective, coaches help coachees reappraise conditions and find new coping strategies (Fontes and Dello Russo, 2021).

#### 5.2.5 Reflection by the coachee enhances resilience

Research shows that reflection by coachees enhances resilience. Coaching provides a space for reflection, allowing coachees to express their concerns and emotions (Lawton-Smith, 2017). Socratic questioning promotes problem-solving and reflection (Neenan, 2018), while this reflective process helps coachees expand their psychological resources (Fontes and Dello Russo, 2021; Hobfoll et al., 2018). Coaches facilitate reflection through questioning, feedback, and insight into strengths and barriers, aiding goal achievement (Gregory et al., 2011). Reflection on personal strengths and limitations fosters resilience (Grant and Kinman, 2014), reduces stress (Grant, 2014), and is crucial for developing psychological capital (PsyCap) (Fontes and Dello Russo, 2021).

### 5.3 Key steps required to deliver effective coaching

#### 5.3.1 A coach should assist the coachee to set goals

A key element in coaching programmes, regardless of the theoretical approach, is goal setting, where the coach assists the coachee in defining objectives and creating an action plan (Fontes and Dello Russo, 2021; Dyrbye et al., 2019). Effective resilience coaching involves setting personally meaningful goals, focusing on the coachee’s current traits and circumstances, and systematically working towards those goals with the coach’s support (Grant, 2014). Coaches help coachees overcome setbacks and customise the program to their unique needs by guiding them in setting personal goals for enhancing resources and resilience (IJntema et al., 2021; Grant, 2014).

#### 5.3.2 A coach should use coaching techniques to guide the coaching process

Facilitating coachee reflection and insight into their strengths and weaknesses is crucial for achieving goals, with coaches using questioning, challenging, and feedback to guide this process (Fontes and Dello Russo, 2021). Instead of presenting information, coaches draw it out from coachees (Neenan, 2018) and help monitor and evaluate progress, providing an intellectual platform for brainstorming and self-reflection (Grant et al., 2009). Reflection helps individuals gain insights into events and generate knowledge for future situations (Jackson et al., 2007). Coaches support coachees in re-examining external conditions and exploring coping strategies (Fontes and Dello Russo, 2021). Socratic questioning clarifies and stimulates coachee thinking (Neenan, 2018). Feedback from coaches fosters resilience by helping coachees identify alternative pathways when facing setbacks (Fontes and Dello Russo, 2021), and positive feedback can enhance psychological capital (Luthans et al., 2006).

#### 5.3.3 A coach should provide a challenging and reflective space

An adequately supported yet challenging environment is essential for developing resilience (Fletcher and Sarkar, 2016; Forbes and Fikretoglu, 2018). Resilience is fostered when coaches facilitate coachee reflection on their thoughts, feelings, and behaviours during sessions (Fontes and Dello Russo, 2021; Jones et al., 2016). By understanding their emotional needs and reactions, coachees learn to cope with adversity (Jackson et al., 2007). Psychological resilience emphasises the connection between an individual’s behaviour, thoughts, and emotions within a specific context (IJntema et al., 2019, 2021). A cognitive-behavioural, solution-focused framework highlights the bidirectional link between thoughts, feelings, behaviours, and the environment (Grant, 2003; Grant et al., 2009). Reflection in coaching raises coachees’ awareness of their current resources and prompts a resource spiral (Hobfoll et al., 2018), encouraging exploration of psychological resource enhancement and identification of potential helpers (Fontes and Dello Russo, 2021). Coaches guide coachees to appraise both internal and external challenges while helping them alter negative emotional and behavioural responses (Neenan, 2018).

### 5.4 The efficacy of the resilience coaching interventions

This scoping review highlighted that participation in coaching consistently improves resilience, corroborating previous research on the efficacy of coaching in resilience development. As coaching research evolves, it incorporates various techniques to build resilience (Grant et al., 2009; Green and Humphrey, 2012; Pemberton, 2015). Coaching has been suggested as a preventive measure for fostering resilience, particularly for individuals facing adversity (Lawton-Smith, 2015). A meta-analysis by Vanhove et al. (2016) found that coaching is more effective at building resilience than traditional classroom-based methods.

Both qualitative and quantitative studies demonstrated positive outcomes in resilience following coaching interventions. Quantitative results, including studies by Dyrbye et al. (2019), Fontes and Dello Russo (2021), and others, showed measurable improvements in resilience. Qualitative feedback also highlighted enhanced resilience, with participants reporting growth in areas like hope, self-efficacy, and stress recovery (Archer and Yates, 2017; Gray, 2016; IJntema et al., 2021).

Several factors explain the success of coaching in improving resilience. First, coaching offers a confidential and proactive space for discussing challenges, which encourages openness (Lawton-Smith, 2015). This emotionally supportive environment promotes cognitive flexibility and learning (Mosteo et al., 2016). Second, coaches help coachees identify patterns and manage emotions, serving as thought partners (Crawford, 2017; Auerbach, 2006). Third, workplace coaching is a tailored, non-hierarchical partnership aimed at fostering goal achievement and wellbeing (Bozer and Jones, 2018; Fontes and Dello Russo, 2021). Through reflective questioning and strategy development, coaches help coachees enhance self-regulation and coping skills, leading to reduced stress and improved resilience (Grant, 2014; Grant and Kinman, 2014).

### 5.5 Measures and instrument currently used to evaluate effectiveness of resilience coaching

This scoping review highlights various tools for measuring resilience.

1. *Single item measure*: A simple Likert-type item, “I recover quickly after stressful experiences,” is used (Auer et al., 2022).
2. Brief resilience scale (BRS): This 6-item tool uses a 5-point Likert scale, including items like “I tend to bounce back quickly after hard times.” Adapted for work-related recovery, it has a 6-point scale where higher scores indicate better recovery (De Haan et al., 2019; IJntema et al., 2021; Song et al., 2020).
3. *Cognitive hardiness scale*: Used in various studies, this scale has 10 to 18 items assessing resilience through cognitive hardiness (Grant, 2014; Grant et al., 2009, 2010, 2017).
4. *Robertson Cooper i-resilience tool*: An online tool that evaluates resilience through four components: Confidence, Purposefulness, Adaptability, and Social Support, providing detailed interpretations (McKimm and Povey, 2018).
5. *Connor-Davidson resilience scale*: This 10-item scale uses a 0–4 scale, with higher scores indicating greater resilience (Dyrbye et al., 2019).
6. *Psychological capital questionnaire (PCQ-12)*: This 12-item tool measures psychological capital, including resilience, on a 7-point Likert scale (Fontes and Dello Russo, 2021).
7. *PCQ (Self-report)*: A 24-item version measuring self-efficacy, resilience, optimism, and hope, using a 6-point Likert scale (Sherlock-Storey et al., 2013).

### 5.6 Additional tools that should be developed or implemented to enhance resilience assessment

To enhance resilience assessment, we encourage coaching programmes to develop tools that will fit the requirements of their programmes. Some studies (e.g., Gray, 2016; Jeannotte et al., 2021) have custom develop scales for their coaching programmes. In the Jeannotte et al. (2021) the nine-dimensional scale set was created and verified after an extensive multiphase examination. The wellbeing model was used in the coaching sessions in Gray (2016) study, and the findings were documented as individual participant coaching profiles. Data on “best self” and “periphery” comments, as well as individual pathway descriptions found during the coaching session, were included in the profiles (Gray, 2016).

We also suggest that it is important to include qualitative measures in order to elicit the views of the coachees on how the coaching programmes have helped to improve their resilience. Semi-structured interviews could be used to gather qualitative data and to establish an in-depth understanding of the participants’ experiences (Archer and Yates, 2017). Participants can also be encouraged to respond to open-ended questions order to gather some qualitative data on participants’ experience of the coaching programmes (Grant, 2014; Grant et al., 2017). Thematic analysis techniques can then be used to analyse the data.

### 5.7 A critical evaluation of the study design elements of the coaching interventions

Theeboom et al. (2014) highlight the importance of research design as a moderating factor in coaching outcomes. The way coaching interventions are studied can influence the results, with more rigorous designs likely to show more reliable outcomes. RCTs are regarded as the “gold standard” for establishing causality because they control for confounding variables and minimise selection bias. While other designs are considered less robust than RCTs due to potential biases (e.g., maturation effects or selection bias), they still offer valuable insights, especially when it is difficult to randomly assign participants.

Athanasopoulou and Dopson (2018) emphasise that studies incorporating multiple data sources—such as multicourse feedback, assessment tools, and repeated measures—are of higher quality than those relying solely on self-reported outcomes. Some of the reviewed coaching studies fall short here, as they rely on subjective evaluations by participants, without triangulating the data through objective measures. Studies that incorporate repeated measures, particularly during and after the coaching process (Rekalde et al., 2017), are better able to track the longitudinal effects of coaching, though few do this comprehensively.

The number of coaching sessions, format, and delivery method (e.g., face-to-face or blended coaching) were found to have no significant effect on outcomes in some studies (Jones et al., 2016; Theeboom et al., 2014). However, Blackman et al. (2016) note that customised, individual programmes tend to yield better results, suggesting that the quality and adaptability of the coaching intervention may be more critical than the number of sessions or the specific delivery format. Moreover, Grover and Furnham (2016) provide evidence supporting the long-term impact of coaching, noting that studies examining coaching effects months after the intervention report sustained positive outcomes. We argue that the lack of longitudinal data in some of the reviewed studies limits our understanding of how long the coaching effects on resilience last and under what conditions they might fade.

The coaching literature is also criticised for its weak theoretical foundation, as many studies lack a clear framework explaining why coaching works (Bozer and Jones, 2018; Grant, 2013; Theeboom et al., 2014). The absence of a unifying approach in coaching for resilience impedes the ability to generalize findings. Bozer and Jones (2018) argue that studies should be evaluated not only on methodological rigor but also on how well they explain the underlying theoretical constructs that make coaching effective.

## 6 Limitations of the study

When extrapolating from the study’s findings, certain limitations need to be taken into account. First, studies that addressed the larger context of resilience coaching were eliminated from our search since it was restricted to English-language literature and only included interventions related to resilience coaching in the workplace. Second, the restricted number of studies included in the review scope limits the scope of this investigation. Third, the study did not include any grey literature, which would have limited the study’s findings. Last, the contextual background of the coaching interventions was restricted to a few settings, which begs the question of whether the findings are applicable in other contexts.

## 7 Agenda for future research

Research on the coaching impact on resilience has not been fully developed yet (Grant, 2017). Psychological resilience is a complex and multi-faceted construct (Grant and Kinman, 2014). The results of this review indicate that personal representations of resilience are extremely varied, and the concept is believed to encompass a wide range of skills and abilities. Notwithstanding some consensus on defining psychological resilience as well as clear findings of its connections to various critical personal outcomes, there is currently no leading, amalgamated theoretical model of individual workplace resilience which is able to be used in all organisational contexts and industries (Rees et al., 2015). Research in resilience coaching still requires a nuanced understanding of its complex elements so as to understand, predict and design suitable interventions in order to improve personal resilience (Liu et al., 2019). Lawton-Smith (2017) suggests a broader conceptualisation of resilience that incorporates both capabilities (skills or strategies) as well as the capacity (more transient resource) for resilience. As resilience encompasses a broad range of abilities and skills and to develop programmes to increase resilience, it is important to understand the competencies that reinforce resilience as well as the strategies that can be employed to improve it, using an evidence-based, rigorous approach (Grant and Kinman, 2014).

More studies are needed on the resilience coaching in order to understand how change occurs during the coaching for resilience process. Resilience has been described as the potential to show resourcefulness through utilisation of available internal and external resources (Pooley and Cohen, 2010). However, Lawton-Smith (2017) suggests that coaching for resilience may have limited impact if it is based only on a defined list of assets, arguing that an attempt to deconstruct, list and quantify a list of attributes may not be an appropriate manner in which resilience can be addressed. She bases her argument on complex adaptive system’s evaluation of a leadership coaching programme (O'Connor and Cavanagh, 2013), where goal achievement, wellbeing as well as transformational leadership behaviours were improved after coaching. A “ripple effect” was shown by that evaluation and secondary gains were clear from the coachees. She then argues that simple linear connections of cause and effect are not adequate to address resilience and wellbeing (Lawton-Smith, 2017). We argue that future research should therefore focus on the change process in resilience coaching interventions and more studies are needed on the resilience coaching approaches, antecedents, distal and proximal coaching results (Mosteo et al., 2016). Moreover, research is needed in order to contribute to better understanding of the dynamic processes under which resilience develops and impacts outcomes in the workplace at various analysis levels. We suggest that this can be achieved by integrating cross-disciplinary understandings of as well as approaches to resilience and resilience coaching so as to have a better understand of the mechanism that enable improved resilience (Rook et al., 2018).

There is a need for more research on how coaching for resilience can respond to an emerging view that resilience should be conceptualised as a dynamic and interactive process whereby an individual experiences adversity and, utilising resources and skills is able to adapt and recover (Kelly et al., 2019). It has been suggested that resilience should no longer be regarded as a static concept but rather as a dynamic process by which people adapt to stressful events or circumstances they are exposed to (IJntema et al., 2019, 2021) as well as positive adaptation within the context of major adversity (Sarkar and Fletcher, 2016). It has also been argued that resilience is fundamentally underpinned by the concept that it is not so much the hard times we face that determine our success or failure as the way in which we respond to those hard times (Jackson and Watkin, 2004). To add further complexity to current discussions of resilience, Fletcher and Sarkar (2016) have argued that a sufficiently supported but challenging environment is required for resilience to develop. What determines the level of resilience is the experiences of the individual, their qualities, as well as each individual’s balance of risk and protective factors (Jackson et al., 2007). There is a need for empirical research to determine and test the various elements in the dynamic process of developing resilience through coaching.

Moreover, consensus is also rising regarding the importance of the environment as well as systemic factors in modern views of resilience (Cusack et al., 2016). The perspective of resilience as a process conceptualises resilience as a function of individuals’ conscious interaction with their external environment (Winwood et al., 2013). Resilience is a process in which the influences of the environment and individuals reciprocally interact, allowing them to adapt, despite the stressors (Menezes et al., 2023). Literature suggests that as an ability, resilience develops over time resulting from many elements that characterise the interaction between an individual and their environment (Baker et al., 2021). Moreover, it has been argued that resilience can be developed and determined by factors that act at the social as well as the individual level, and that the environment in which an individual must survive may support or undermine their personal resilience (Howe et al., 2012). Research is needed to see how coaches help coachees to navigate this interaction between the individual and their external environment as part of the coaching process.

The impact of biological and genetic factors is an area of resilience coaching research that needs further exploration. It has been suggested that psychological resilience entails the interaction between cognition, behaviour, as well as affect in a specific time and context (IJntema et al., 2019). The “fourth wave” of resilience research places more emphasis on a focus on multifaceted dynamics and processes that connect genes, brain development, neurobiological adaptation, behaviour as well as context at various levels (Wright et al., 2013). More research in resilience coaching is needed in order to understand the impact of the interaction between cognition, behaviour, time and context as multifaceted factors in achieving positive resilience coaching outcomes.

The importance of social support in enhancing psychological resilience, particularly in the context of coaching for resilience requires further inquiry. Research has demonstrated that one of the fundamental elements of resilience is social support, and maintaining relationships is an element of social support (Jackson et al., 2007). It has been posited that resilience can be built and determined by elements that operate at both individual and social levels, and that the context whereby a person must survive may provide support or undermine their individual resilience (Howe et al., 2012). Empirical research is needed to explore how coaches can integrate social relationships in assisting coachees to improve resilience.

To help to develop a more effective coaching approach, future research studies should also explore how cross-disciplinary insights into resilience can inform coaching practices. Despite the widespread use of the term “bounce-back,” its exact meaning—whether it refers to emotional stability, performance, or something else—remains unclear (Lawton-Smith, 2017). Neenan (2018) critiques the notion that resilience merely involves returning to a pre-adversity state, arguing it oversimplifies the complex emotional struggles individuals may face during recovery. Resilience is fundamentally about how individuals respond to adversity rather than the adversity itself (Jackson and Watkin, 2004). This perspective suggests that resilience should not be framed merely as bouncing back; instead, it should encompass the appraisal of both adverse events and one’s ability to manage them (Baker et al., 2021). Conceptualising resilience in terms of sustainability may be more relevant to coaching, emphasizing the importance of resilience in both current and future contexts (Lawton-Smith, 2017). Oversimplifying resilience as a return to a previous state risks overlooking the transformations, growth, and learning that occur following challenging experiences (Crawford, 2017). Thus, a critical goal of resilience coaching should be to establish a balance between encountered adversities and the available resources. Future research in resilience coaching should focus on this balance, exploring how coaches can effectively support coachees in navigating challenges while fostering growth and resilience (Skews et al., 2018).

The fragmented nature of the literature, reliance on self-reporting, and inconsistent methodological rigor limit the generalizability of the findings. Nevertheless, studies with stronger designs—such as those that include control groups, pre/post measures, and theoretical frameworks—offer valuable insights into the efficacy of coaching. Future research should aim to integrate more robust theoretical models, longitudinal data, and larger, more diverse samples to build a more coherent understanding of coaching’s long-term impacts.

## 8 Conclusion

This scoping review shows that coaching is a useful intervention for enhancing resilience and helping people who are experiencing adversity. Specifically, the scoping review highlighted three crucial aspects. First, the scoping review demonstrated that a variety of coaching philosophies can be effectively employed to support resilience coaching programmes. Second, the scoping review highlighted various factors that are key in coaching for psychological resilience, including: goal setting; the creation of coachee resilience resources; the coach-coach relationship; the coach’s role as helping coachees go through the self-regulation cycle; and the importance of the coachee working between coaching sessions. Third, the scoping review demonstrated the efficacy of coaching interventions as participation in coaching resulted in improved resilience for the participants in the coaching interventions. However, the scant number of studies that surfaced from the literature search indicates that more study on resilience coaching is required. Although coaching theories, research, and practice that focus on coachees gaining resilience through a range of strategies and tools are growing, research on the influence of coaching on resilience has not reached its full scope.
